# The effect of smartwatch head shape on visual imagery perception

**DOI:** 10.1371/journal.pone.0290259

**Published:** 2023-08-24

**Authors:** Yu-Liang Feng, Yang-Cheng Lin, Chun-Chin Chen

**Affiliations:** 1 School of Art and Design, Jiangsu University of Technology, Jiang Su, China; 2 Department of Industrial Design, National Cheng Kung University, Tainan, Taiwan; 3 Department of Industrial Design, National Kaohsiung Normal University, Kaohsiung, Taiwan; Southern Taiwan University of Science and Technology, TAIWAN

## Abstract

Obesity-related diseases have been on the rise, making it important to promote physical activity. Smart sports watches are popular among young people and can play a role in this regard. This study aims to evaluate the impact of different watch head design types on the visual image of smart sports watches. Based on sales data, seven sports smartwatches with sales of over 2000 units were selected from a sample of 50 as representative samples. A factor analysis and questionnaire survey were used to identify four groups of adjectives that describe watch heads: Sporty and Smart, precious and exquisite, distinctive and avant-garde, and trendy and technological. College students evaluated the seven watches using these adjectives, and using triangular fuzzy mathematics theory, the watches were divided into three categories. The results show that the seven watches had significant differences in appearing "Sporty and Smart" and "precious and exquisite", while the visual imagery of "distinctive and avant-garde" and "trendy and technological" had no significant difference. Based on the grouping analysis of the seven samples, it is concluded that: the slim and compact shape without excessive decoration has a sense of sportiness and simplicity; the square shape combined with left and right buttons has a sense of sportiness and fashion; the unique connection between the round shape, the watch strap, and the watch head, as well as the strong mechanical feeling, have a sense of value. To substantiate the validity of our research findings, we devised three novel specimens based on the morphological elements of sports watches and conducted surveys accordingly. Statistical analysis revealed a fundamental coherence between the performance of these specimens in four stylistic domains and the expression of style-forming elements, confirming the reference value of these findings in the stylistic design of sports smartwatches. This study provides designers with references for improving the design and development efficiency of smart sports watches, promoting their sustainable development.

## Introduction

Wearable devices have become increasingly popular in various fields due to the rapid development of the Internet and wearable intelligent interactive technology. Wearable devices have important applications and research value [1–3]. The World Health Organization (WHO) survey report in 2000 shows that 850,000 people in developed countries die every year due to a lack of physical exercise [4]. In recent years, the prevalence of overweight and obesity in China has increased rapidly and has become a pressing public health issue [5]. Obesity and overweight can lead to chronic diseases such as hypertension, diabetes, and cardiovascular disease [6, 7]. Exercise helps prevent chronic disease and has a positive effect on reducing human mortality [4, 8, 9]. Previous studies have shown that sports equipment with AI exercise programs can increase enthusiasm for exercise [10, 11]. Smart sports watches are currently the most widely used smart wearable devices in the sports field [12, 13]. In product design, meeting the basic functional needs of users is essential. However, it is also important to consider users’ demands, expectations for new product functions, and expectations for styling. The external manifestation of consumers’ own needs and preferences is consumption behavior [14]. Consumer psychological cognition research has shown that the appearance of products is extremely important among the factors affecting purchase decisions [15]. Baxter’s five senses research on imagery found that visual images had the greatest impact on imagery perception [16].

Previous studies on the design of smart bracelets have mainly focused on shape design. Zhang explored the modeling design of female users’ smart bracelets, producing innovative designs for the appearance and some functions of smart bracelets from the perspectives of female users’ consumption psychology, lifestyle, and aesthetic characteristics [17]. Zhu studied the appearance of smart products from the perspective of user-centered personalization, creative innovation, and cultural characteristics, and proposed new design ideas [18]. Zhao et al. studied the image characteristics of smart watches from the perspective of urban health management and explored the preference characteristics of different groups [19]. In ergonomics research, Wang (2014) analyzed and designed the interface interaction, function, and size of smart watches. Yang evaluated and modeled an age-appropriate smart bracelet using the FAHP and TOPSIS methods from four dimensions: product design, human–computer interaction, user experience, and actual production [20]. Ding (2019) applied the theory of Kansei Engineering to the study of smart wearable devices, exploring the extraction of user’s perceptual demand factors and design factors and applying it to the establishment of a design and development model of smart bracelets [21]. Yeh and Lin (2006) demonstrated the advantages of using neural network models in product form design, enabling product designers to understand consumer perception and incorporate emotions into design elements [22].

Although many researchers have conducted related studies on smart watches from the aspects of user experience, behavior, and disease-related effects [23–29], most of these studies are market-oriented, and few have focused on visual evaluation and users’ psychological feelings. Hence, this study seeks to explore the visual imagery of smartwatches from the perspective of user’s visual perception, employing factor analysis and fuzzy theory to analyze the visual imagery of smartwatches, thereby offering valuable design insights to designers.

## Materials and methods

### Research framework

**Fig 1:**
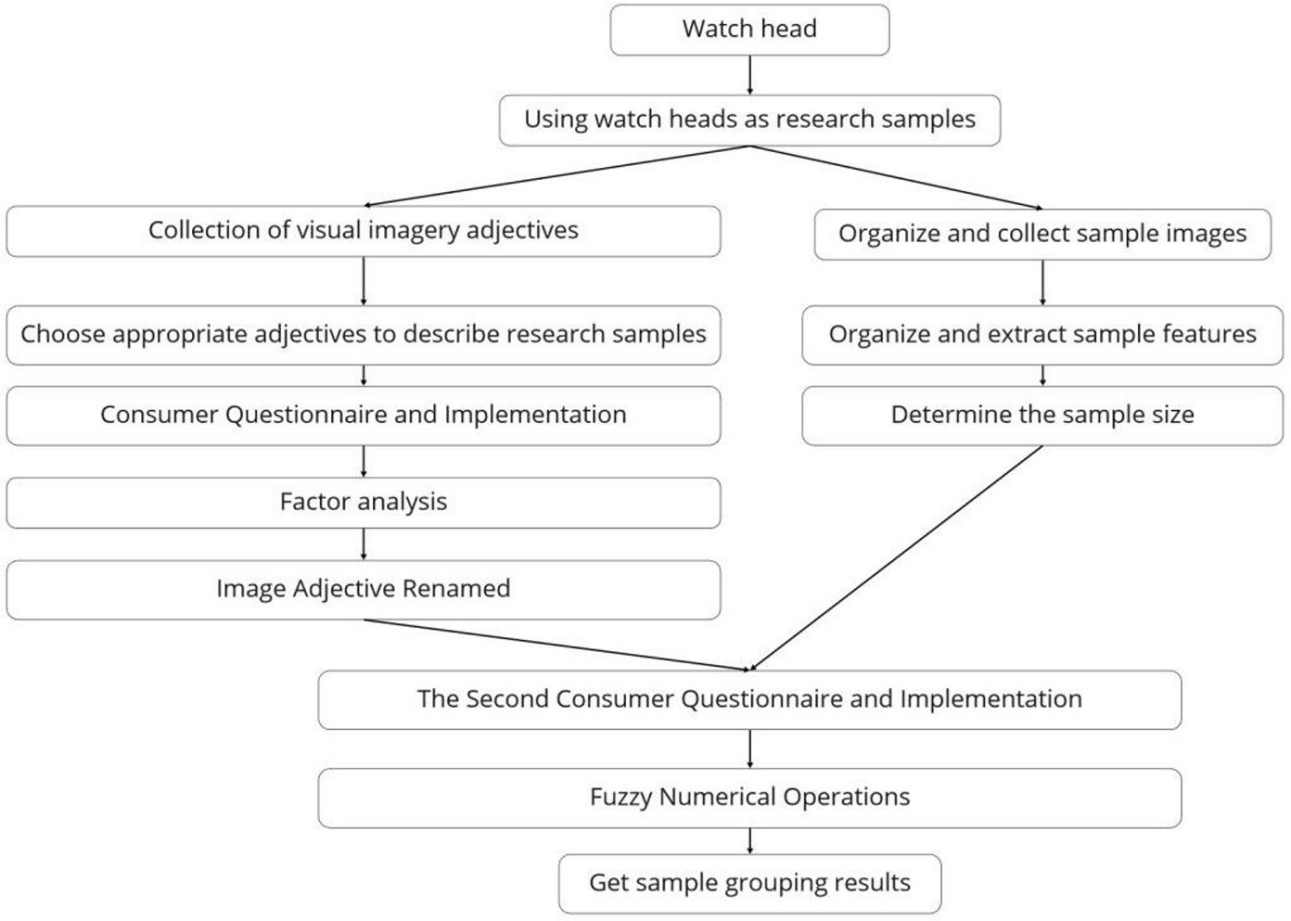
Research process.

We are set to explore the visual imagery associated with the head shapes of smartwatches, employing questionnaires, factor analysis, and fuzzy logic as tools. The research process is depicted in a schematic form in Fig 1, with a detailed outline of the steps to execute the study.

**Step 1**: We compiled a list of adjectives that are suitable for describing the visual imagery of the watch head shape. The adjectives were collected through three methods. Method 1: We collected published papers from online databases. Method 2: We gathered information from online sales platforms, such as publicity content and user evaluations (Taobao, Jingdong, Suning). Method 3: We conducted interviews with industry experts and high-involvement groups to gather important information. From the collected adjectives, we selected 100 words that were appropriate for describing the appearance of watch heads. The selected adjectives are listed in Table 1.

**Table 1:** The 100 adjectives that describe the sample smart sports watches.

**business style**	**traditional**	**technological**	**sporty**	small	hardy	cryptic	form	smoothly	**personalized**
**rounded**	soft	**graceful**	**telegraphic**	smooth	soft-edged	decorative	sophisticated	comfortable	pure
Featured	cute	perceptual	**future sense**	**rigorous**	offbeat	retro	**avant-garde**	lively	**modern**
**elegant**	cordial	**active**	energetic	**geometric**	fusion	artistic	**charismatic**	**high-end**	useful
**hard**	lavish	understated	**stylish**	naughty	flexible	elegant	serious	**lightweight**	clean
flavorful	**delicate**	slim	charming	**high quality**	low cost	exaggerated	commemorative	beautiful	strange
rustic	fantastic	feminine	handsome	**innovative**	**tasteful**	**integrated**	**advanced**	peculiar	generous
striking	manly	**valuable**	exquisite	dignified	**unique**	fresh	thin	heavy	harmonious
**big watch dial**	**beautiful**	**cool**	**smart**	**high-tech**	**electronic**	**scientific**	engineering male style	**multifunctional**	square
**mechanical**	**expensive**	high quality	mature	international	**trendy**	**stable**	eye-catching	cute	hot

**Fig 2:**
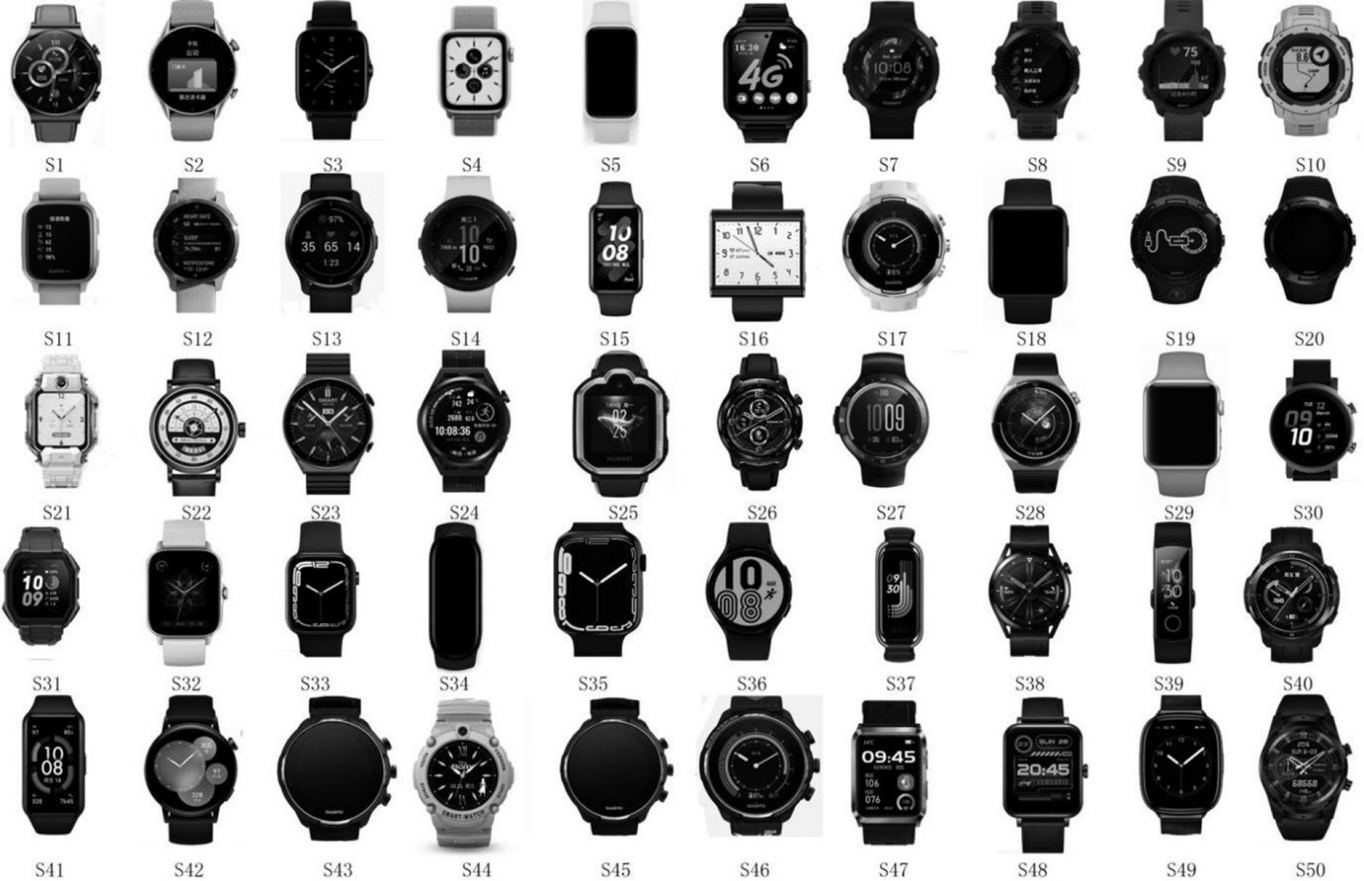
The 50 collected watch samples.

**Fig 3:**
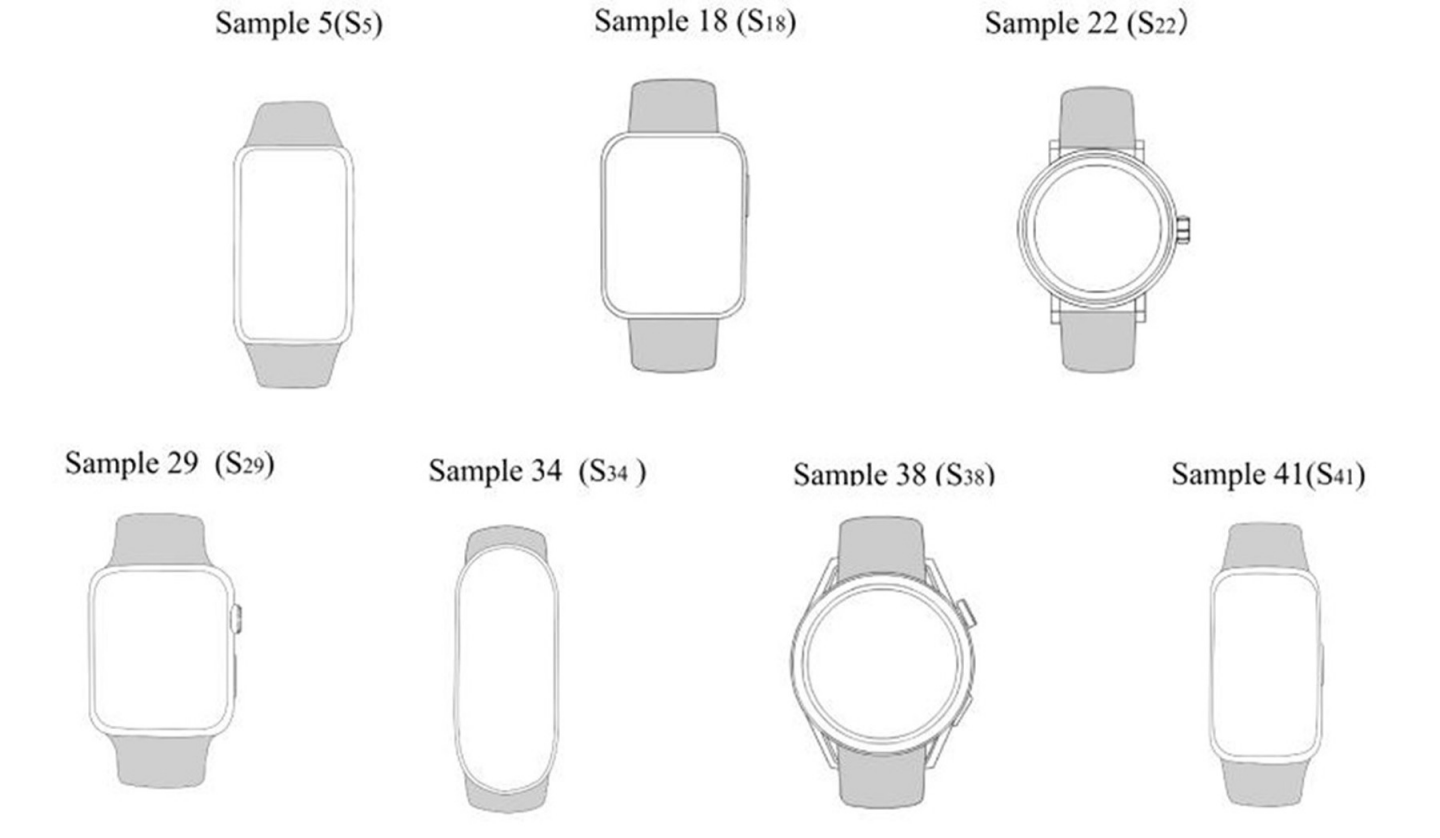
Study sample grayscale image.

**Step 2:** Initially, we collected 200 exemplary smartwatch images from various sources, including dedicated smartwatch retailers in shopping centers, assorted online sales platforms, and comprehensive electronics periodicals. Subsequently, we engaged the expertise of ten experienced sports watch users to carefully evaluate the collected watches and narrowed down the selection to a widely-used subset of 50, as shown in Fig 2. To further refine and enhance the universality of our samples, we opted for those smart sports watches on the Taobao platform with a monthly sales volume exceeding 2000 units. The eighth-ranked watch demonstrated a monthly sales average below 1500 units; thus, we elected to focus on the seven smartwatches ranked within the top seven in terms of sales as our final subjects of study, as illustrated in Fig 3.

**Step 3:** We processed the sample images to eliminate the influence of other design factors and highlight the shape of the watch heads. In order to achieve this, we used Photoshop drawing software to convert the sample images to grayscale, delete or simplify the color, texture, background, and straps of the watches, as shown in Fig 3.

**Step 4:** In order to clarify the adjectives corresponding to the appearance of the smart sports watch head, we used the questionnaire survey method and the factor analysis method to further select the descriptors. First, five designers, three college professors, and two PhD experts in product design were invited as the subjects to be tested. Second, they were asked to select 30 to 40 adjective words from the 100 adjectives that fit the visual image of the watch head according to their subjective feelings. Subsequently, 36 adjectives with more than 6 occurrences were sorted out (Table 1, bold font). In addition, a set of semantic difference questionnaires were developed by sorting out the thesis data and expert opinions to clarify the similarities and affiliations among the 36 adjectives. The questionnaire design was based on the Likert five-point scale method, and the subjects chose five different levels according to different feelings: "very unsuitable", "unsuitable", "average", "suitable", and "very suitable". Then, the questionnaires were distributed and returned, and the data analysis of the questionnaires was carried out by means of factor analysis. Finally, a total of 122 valid questionnaires were collected, including 64 from male participants and 58 from female participants. Then, according to the analysis results, each type of adjective was classified and named.

**Step 5:** We used the questionnaire survey method and triangular fuzzy mathematical calculation to realize the objective evaluation of the visual image of the smart sports watches. First, potential users of smart sports watches were invited to complete a questionnaire survey on seven samples (see Fig 3). Before filling in the questionnaire, the testers viewed the sample pictures in sequence from 1 to 7, evaluated each sample according to their real feelings, and filled in the questionnaire. Ultimately, the questionnaire data underwent a triangular fuzzy operation to ascertain the cumulative utility value for each sample, after which a radar graph was constructed. In the end, there were 294 questionnaires deemed valid, comprising 150 from males and 144 from females.

### Factor analysis

Factor analysis is a statistical method that allows researchers to identify underlying factors that contribute to the variation observed in a set of variables [30]. By reducing the dimensionality of the variables, factor analysis helps to uncover the essential characteristics of the phenomenon being studied [31]. In the context of bicycle image design, factor analysis can help to simplify complex factors, discover the intrinsic nature of the design elements, and identify essential features [32, 33]. The process of conducting factor analysis involves several steps: (1) determining the analysis content index; (2) identifying the relationship criterion through the analysis of the relationship between the indicators and factors; and (3) estimating the factor load of each factor using a model, which allows researchers to determine the degree of influence of each factor [34]. In this study, we employed factor analysis to transform users’ visual perception into quantitative data and obtained visual image features [32].

### Fuzzy logic

Fuzzy logic, proposed by Zadeh in 1965, is a mathematical method that deals with uncertainty and imprecise information in human language [35–37]. The theory of ambiguity delineates and characterizes the relationship between elements and sets through membership degrees within the confines of the interval [0,1]. Let ∪ denote the universal set, with μ acting as a function, that is, μ: ∪→ [0,1]. The membership function of the fuzzy subset *A* is thus represented as μ_A_(X), implying the degree of affiliation of element x within the fuzzy set *A*. In the case of discretization, the fuzzy set *A* can be articulated as follows:

$$A=\frac{\mu_{A}\left(x_{1}\right)}{x_{1}}+\frac{\mu_{A}\left(x_{2}\right)}{x_{2}}+\cdots +\frac{\mu_{A}\left(x_{n}\right)}{x_{n}}$$

In the context of continuity, however, the fuzzy set *A* is portrayed as follows:

$$A=\int_{x\in U}\frac{\mu_{A}(x)}{x}$$

**Fig 4:**
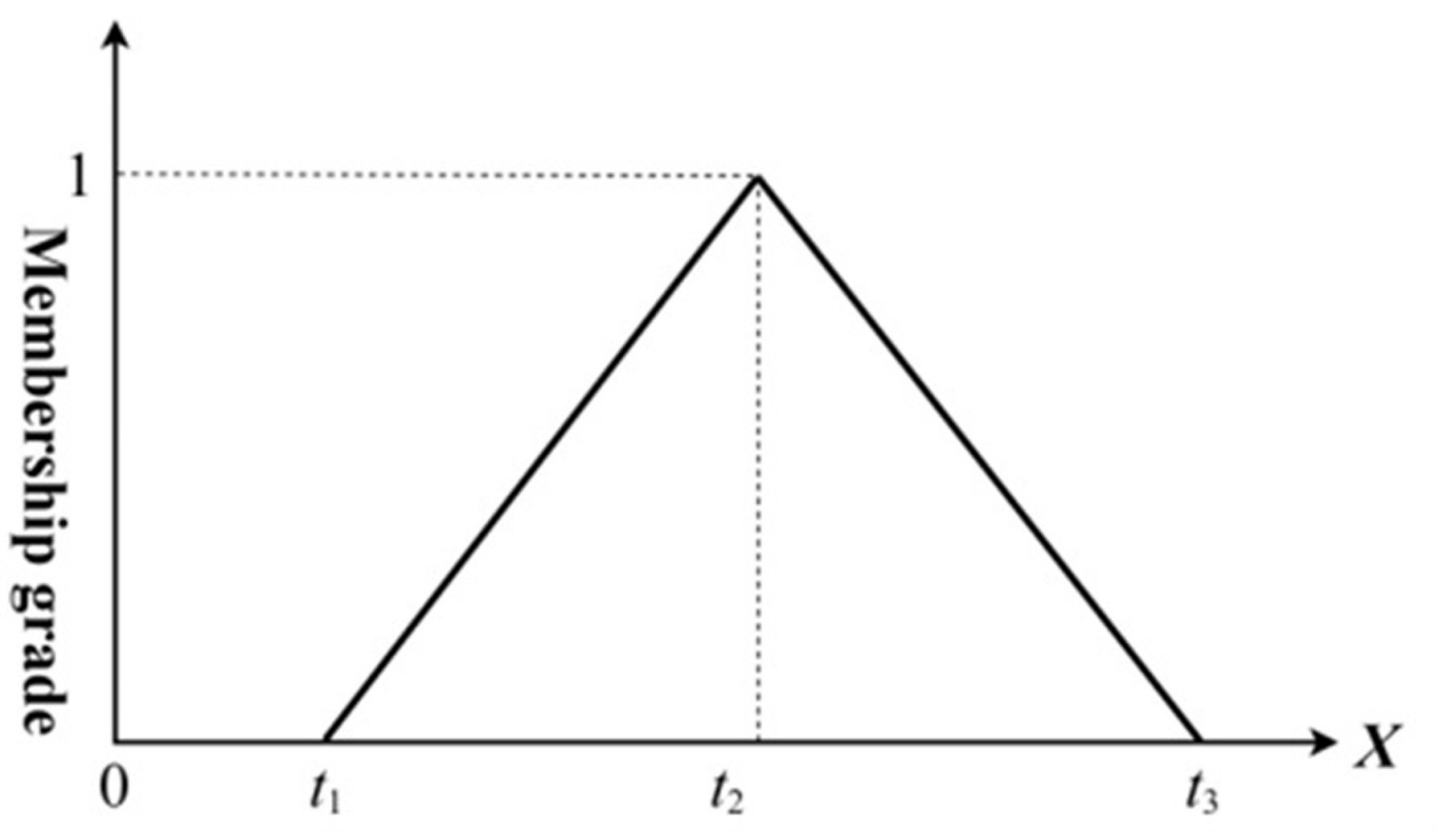
The membership function of a triangular fuzzy number.

Fuzzy logic allows for the conversion of vague and uncertain linguistic terms into quantitative values through scientific operations [38]. In fuzzy logic, fuzzy numbers are employed to quantify semantics and they typically encompass triangular fuzzy numbers, trapezoidal fuzzy numbers, and normal fuzzy numbers. Triangular fuzzy numbers are the most commonly used, characterized by their membership function distribution in the shape of a triangle [39], as shown in Fig 4.

Triangular fuzzy number: This is a widely adopted type of fuzzy number, characterized by its triangularly distributed membership function. Defined by three parameters (*t*_*1*_, *t*_*2*_, *t*_*3*_), the triangular fuzzy number possesses *t*_*2*_ as its apex, with *t*_*1*_ and *t*_*3*_ denoting the support on the left and right respectively. Given the simplicity and comprehensibility of such fuzzy number computations, it enjoys widespread acceptance in myriad practical scenarios [40]. A triangular fuzzy number can be depicted as $\tilde{A}$ = (*t*_1_, *t*_2_, *t*_3_), with its corresponding membership function and graphical representation as follows:

$$\mu_{\tilde{A}}(x)=\left\{\begin{array}{cc} 0 & ,\;\;\;\;\;\;\;\;x<0\\ \left(x-t_{1}\right)/\left(t_{2}-t_{1}\right) & ,\;\;\;t_{1}\leq x\leq t_{2}\\ \left(t_{3}-x\right)/\left(t_{3}-t_{2}\right) & ,\;\;\;t_{2}\leq x\leq t_{3}\\ 0 & ,\;\;\;\;\;\;\;\;x\geq t_{3} \end{array}\right.$$

**Fig 5:**
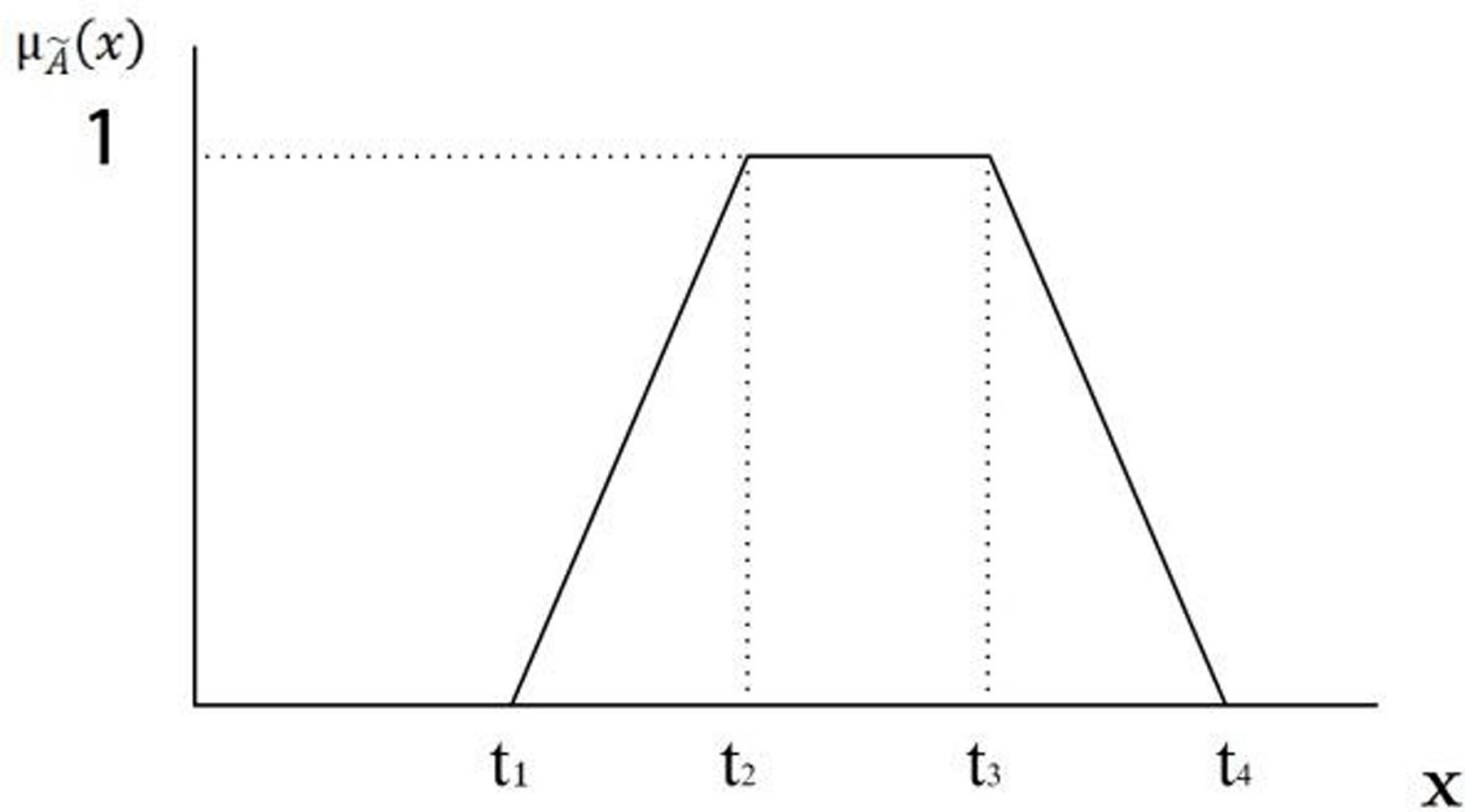
Membership functions of trapezoidal fuzzy numbers.

Trapezoidal fuzzy number: This is another common type of fuzzy number, distinguished by its trapezoidal distribution of the membership function. Represented by four parameters (*t*_*1*_, *t*_*2*_, *t*_*3*_, *t*_*4*_), the trapezoidal fuzzy number identifies *t*_*1*_ and *t*_*4*_ as the support on the left and right respectively, with *t*_*2*_ and *t*_*3*_ delineating the interval with a membership of 1. Compared to triangular fuzzy numbers, trapezoidal fuzzy numbers provide a superior representation of uncertainty with stable intervals [37]. The trapezoidal fuzzy number can be denoted as $\tilde{A}$ = (*t*_1_, *t*_2_, *t*_3_), with its respective membership function definition and graphical representation (Fig 5) as follows:

$$\mu_{\tilde{A}}(x)=\left\{\begin{array}{cc} 0 & ,\;x<t_{1}\\ \left(x-t_{1}\right)/\left(t_{2}-t_{1}\right) & ,\;t_{1}\leq x\leq t_{2}\\ 1 & ,\;t_{2}\leq x\leq t_{3}\\ \left(t_{4}-x\right)/\left(t_{4}-t_{3}\right) & ,\;\;\;\;\;t_{3}\leq x\leq t_{4}\\ 0 & ,\;\;\;\;\;\;\;\;\;\;x\geq t_{4} \end{array}\right.$$

**Fig 6:**
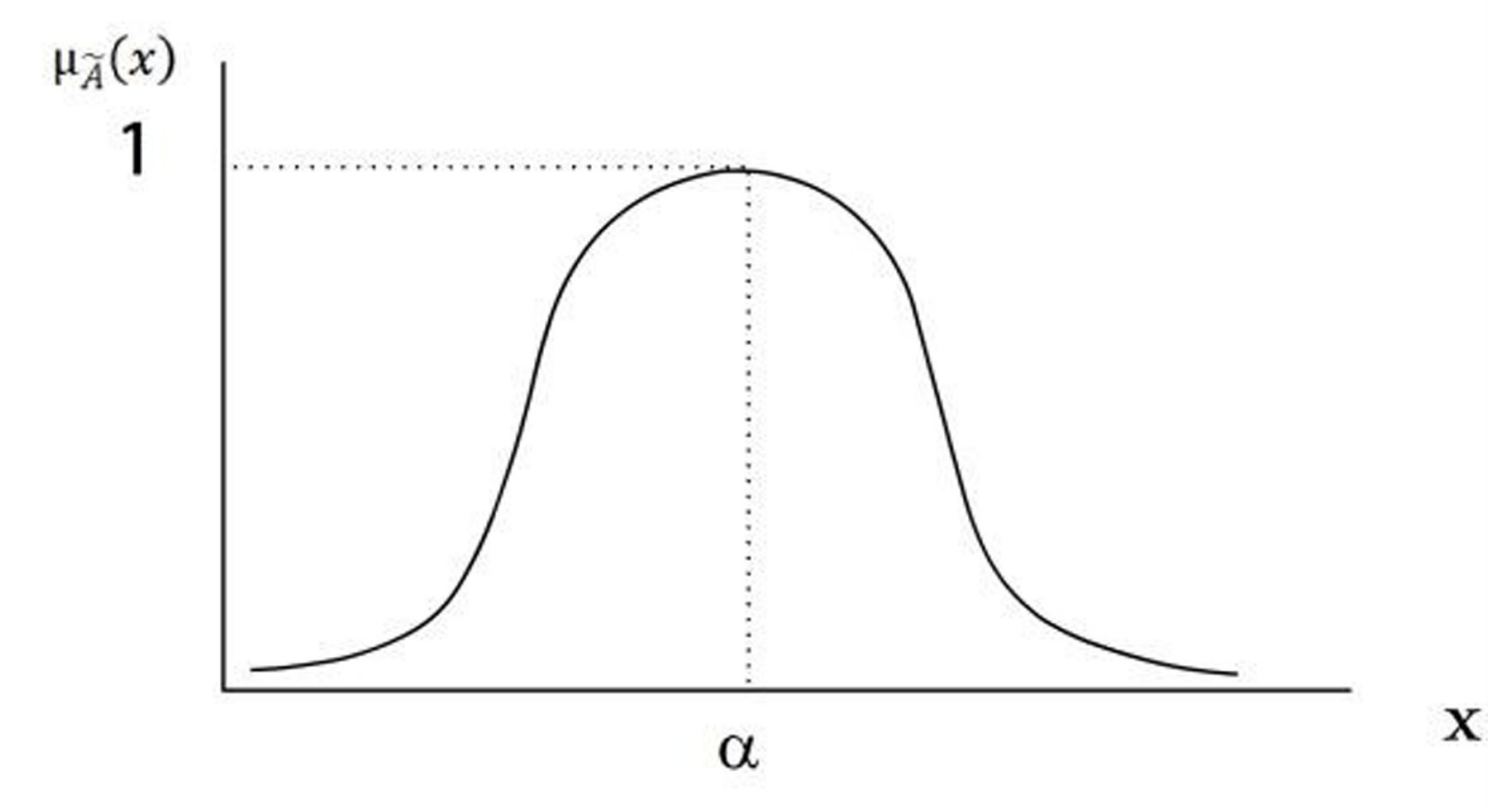
Membership function of normal fuzzy numbers.

Normal fuzzy number: The membership function distribution of a normal fuzzy number takes the form of a normal or Gaussian distribution. A normal fuzzy number is represented by two parameters (*μ*, *σ*), where *μ* is the mean and *σ* is the standard deviation. The normal fuzzy number aptly describes uncertainty in the natural world as numerous phenomena adhere to a normal distribution [37]. Its membership function definition and membership function graphical representation (Fig 6) are as follows:

$$\mu_{\tilde{A}}(x)=e^{-{\left(\frac{x-a}{b}\right)}^{2}},\cdot \text{b}>0$$

In assessing the visual image of the face of a smart sports watch, the triangular fuzzy number is chosen over the trapezoidal or normal fuzzy number, primarily due to its simplicity, intuitiveness, and practicality. It can be succinctly described with just three parameters (lower bound, most likely value, upper bound), rendering it easy to compute. Both its shape and membership function provide an intuitive reflection of the extent and degree of uncertainty, making it easy to understand and interpret. In previous research, such as those conducted by Hsiao (2013), Lin (2018), and Wu (2022) on bicycle wheelset styling imagery and bicycle frame design, the triangular fuzzy number has proven its efficient performance in dealing with fuzzy and uncertain information, satisfying the requirements of this study [33, 41, 42]. Zheng and Lin (2017) incorporated triangular fuzzy numbers in the design of a fuzzy TOPSIS expert system in neural networks and created perfume bottles with different style values [43]. Lin et al. (2007) introduce a novel fuzzy logic methodology for identifying the optimal amalgamation of form elements in mobile phone design that best aligns with a given product image [44]. Therefore, the triangular fuzzy number is chosen for this research.

In our study, we used a similar method to Wu et al. and Chen et al. by using triangular fuzzy numbers to evaluate the visual imagery of smart sports watch heads. We also utilized linguistic variables to design a perceptual evaluation questionnaire and obtain the affiliation of each sample with specific adjectives. Our approach allowed us to objectively evaluate and compare the visual imagery of different smart sports watch head designs. Additionally, our study contributes to the existing literature on the use of fuzzy logic in design evaluation and further demonstrates the potential of this method in various design fields.

**Fig 7:**
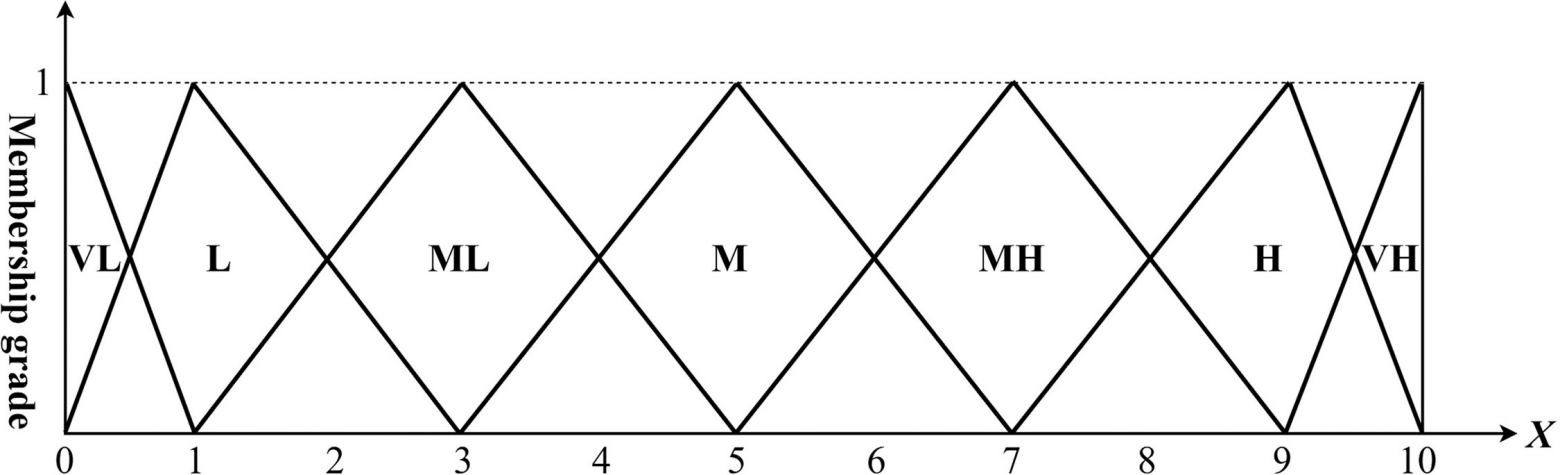
Triangular fuzzy number function.

To determine the user’s evaluation of the visual imagery of the watch heads, a perceptual evaluation questionnaire was designed using linguistic variables such as "very low," "low," "medium low," "medium," "medium high," "high," and "very high" as the evaluation scale. Table 2 presents the questionnaire. Fig 7 illustrates how triangular fuzzy numbers can be used to denote the degree of affiliation a research sample has with any given adjective.

**Table 2:** Linguistic variables for assessing importance and ratings.

Linguistic Variables	Triangular Fuzzy Numbers
Very low	(0, 0, 1)
Low	(0, 1, 3)
Medium low	(1, 3, 5)
Medium	(3, 5, 7)
Medium high	(5, 7, 9)
High	(7, 9, 10)
Very high	(9, 10, 10)

Taking the S22 sample as an instance, for instance: when the subject observes a round sports watch with a direct link bracelet, and scores it 6 points under the category of Precious-Exquisite, it translates into the fuzzy number (7, 9, 10). Again, employing the S34 sample as an example, when the subject encounters wristwatches whose form is svelte and compact, devoid of any superfluous decorative structure, evoking a potent impression of athleticism and simplicity, and rates it 7 points in the Sporty-Smart criterion, it translates into the fuzzy number (9, 10, 10).

### The operation rules of total utility value

Defuzzification is the process of converting fuzzy values to crisp values. Common defuzzification methods include the minimum set method and the maximum set method, which compare the triangular fuzzy numbers in the membership function and interpret them as definite values [45]. In this study, we calculated the total utility value of the visual image of smart sports watches using both the minimum set and maximum set methods. The calculation can be expressed as follows:

Assuming there are n triangular fuzzy numbers in the membership function of the triangular fuzzy numbers, ${\tilde{t}}_{\text{i}}$ = (*t*_*i*1_, *t*_*i*2_, *t*_*i*3_), *i* = 1,2,……,n, the minimum membership function *μ*_*G*_(*x*) and the maximum membership function *μ*_*M*_(*x*) can be expressed as *G* and *M*, respectively, *G* = (*X*_*min*_,*X*_*min*_,*X*_*max*_) and *M* = (*X*_*min*_,*X*_*max*_,*X*_*max*_). Therefore, the total utility value *U_T_*(${\tilde{t}}_{\text{i}}$) of triangular fuzzy numbers can be expressed as:

$$U_{T}\left({\tilde{t}}_{i}\right)=\left[\begin{array}{c} \left(t_{i3}-X_{min}\right)/\left(\left(X_{max}-X_{min}\right)+\left(t_{i3}-t_{i2}\right)\right)+1\\ -\left(X_{max}-t_{i1}\right)/\left(\left(X_{max}-X_{min}\right)+\left(t_{i2}-t_{i1}\right)\right) \end{array}\right]/2,i=1,2,\ldots ,n$$

To better understand users’ perceptions of the visual imagery of seven smart sports watches, we designed an evaluation questionnaire that used semantic variables and renamed adjectives. We calculated the total utility value of each watch head for each visual image adjective using the equation and created a radar chart.

## Results and discussion

In this study, we invited five designers, three college professors, and two PhD experts in product design to screen out 36 adjectives that appeared more than six times from the 100 adjectives that were suitable for describing the image of smart sports watches. Using these 36 adjectives, we designed a questionnaire and conducted a survey. Finally, a total of 122 valid questionnaires were collected, including 64 from male participants and 58 from female participants. Subsequently, we used SPSS 20 software to conduct factor analysis on the questionnaire data, which yielded the following results.

We performed factor analysis on the 36 adjectives with a discriminative degree. After principal component analysis, we screened out factors with absolute values of factor loading greater than 0.6, resulting in the selection of 22 adjectives. We then performed another factor analysis, as shown in Table 3.

**Table 3:** Absolute values of factor loadings for 22 adjectives.

Adjectives	Initial	Extraction	Adjectives	Initial	Extraction
energetic	1	0.736	trendy	1	0.74
sporty	1	0.796	integrated	1	0.764
telegraphic	1	0.814	multifunctional	1	0.796
future sense	1	0.789	golden	1	0.684
smart	1	0.826	rigorous	1	0.619
elegant	1	0.737	hard	1	0.641
big watch dial	1	0.747	mechanical	1	0.714
innovative	1	0.801	delicate	1	0.689
tasteful	1	0.808	scientific	1	0.675
unique	1	0.734	high-end	1	0.693
electronic	1	0.814	square	1	0.609

### Reliability analysis and factor analysis

The analysis begins by assessing the internal consistency of the questionnaire items with Cronbach’s Alpha, which is found to be 0.964. This value is remarkably close to 1, implying a high level of internal consistency. It signifies that the items are effectively measuring a cohesive construct.

Subsequently, the suitability of the data for factor analysis is evaluated through the Kaiser-Meyer-Olkin (KMO) measure. With a value range of 0 to 1, a KMO value closer to 1 indicates greater suitability for factor analysis. In this dataset, a KMO value of 0.933 signifies excellent suitability. To further establish the appropriateness for factor analysis, Bartlett’s Test of Sphericity is employed to ascertain if there are significant correlations among the variables. With a chi-square value of 3069.273, degrees of freedom at 351, and a significance level of 0.000, there is strong evidence of adequate correlations among the variables.

Delving into the results of the factor analysis, the first factor accounts for a substantial 56.941% of the variance, suggesting it might represent a pivotal underlying dimension. The second factor contributes an additional 8.547%, bringing the cumulative variance to 65.489%. Similarly, the third factor adds 3.9%, and the fourth factor adds 3.425%, culminating in a total explained variance of 72.813%. This is noteworthy, as surpassing 70% is generally regarded as commendable. Moreover, all four factors have eigenvalues greater than 1, which can be observed in Table 4.

**Table 4:** Total explained variance.

Component	Initial Eigenvalues			Square Loading Extraction			Transformed Square Loading		
	Total	Variance	Accumulative	Total	Variance	Accumulative	Total	Variance	Accumulative
		(%)	(%)		(%)	(%)		(%)	(%)
1	19.36	56.941	56.941	19.36	56.941	56.941	7.312	21.507	21.507
2	2.906	8.547	65.489	2.906	8.547	65.489	6.701	19.709	41.216
3	1.326	3.9	69.389	1.326	3.9	69.389	5.603	16.481	57.697
4	1.164	3.425	72.813	1.164	3.425	72.813	5.14	15.117	72.813

The total explained variance was 72.813%, and all four factors had eigenvalues greater than 1, as shown in Table 4. The transformed matrix in the factor analysis, as shown in Table 5, indicates that the factors of four Factors are distinct and other factors are not involved. Consequently, the factor analysis resulted in the identification of 22 adjectives and four components for further research.

**Table 5:** Transformed matrix.

Adjectives	Component			
	Factor 1	Factor 2	Factor 3	Factor 4
sporty	0.82	0.1	0.2	0.272
telegraphic	0.801	0.119	0.377	0.125
energetic	0.793	0.289	0.092	0.125
smart	0.782	0.173	0.234	0.36
future sense	0.718	0.193	0.422	0.239
multifunctional	0.638	0.164	0.258	0.543
mechanical	0.067	0.796	0.258	0.098
big watch dial	0.145	0.764	-0.051	0.374
golden	0.297	0.754	0.167	0.01
hard	0.248	0.697	0.234	0.197
elegant	0.187	0.69	0.475	-0.005
square	0.163	0.662	0.23	0.3
high-end	0.166	0.654	0.438	0.214
delicate	0.281	0.61	0.475	0.105
tasteful	0.266	0.441	0.677	0.292
innovative	0.466	0.219	0.643	0.351
rigorous	0.291	0.328	0.632	0.166
unique	0.234	0.372	0.623	0.392
electronic	0.295	0.192	0.163	0.815
integrated	0.271	0.285	0.237	0.744
scientific	0.325	0.155	0.384	0.631
trendy	0.328	0.239	0.449	0.611

### Renaming results of factor analysis

After screening 36 adjectives through factor analysis, 22 adjectives belonging to four groups were obtained. To better reflect the meaning of each group, the adjectives within each group were renamed. According to Table 5, the renamed adjectives are Sporty and Smart, Precious and Exquisite, Distinctive and Avant-garde, and Trendy and Technological.

**Table 6:** Renaming of new factors.

Factor	Adjective groups	Factor naming	Code
Factor 1	sporty; telegraphic; energetic; smart; future sense; multifunctional	Sporty–Smart	S&S
Factor 2	mechanical; big watch dial; golden; hard; elegant; square; high-end; delicate	Precious–Exquisite	P&E
Factor 3	tasteful; innovative; rigorous; unique	Distinctive–Avant-garde	D&A
Factor 4	electronic; integrated; scientific; trendy	Trendy–Technological	T&T

### Assessment of visual imagery of watch heads

**Fig 8:**
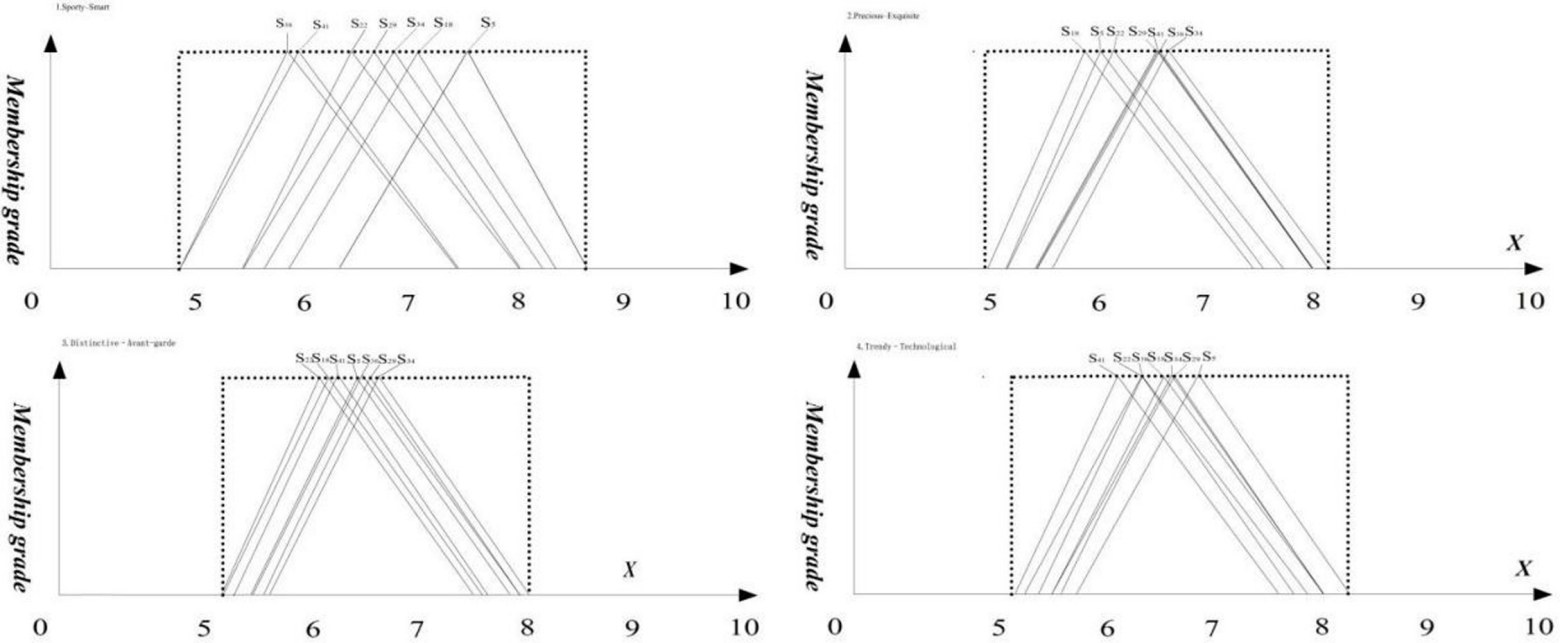
The seven distinct watch head types’ triangular fuzzy numbers in each visual assessment.

To assess the visual imagery of the seven watch heads, we conducted a questionnaire survey using semantic variables (refer to Table 2) of the four renamed groups of adjectives on a sample of seven watch heads (refer to Fig 3). A total of 294 valid questionnaires were collected, comprising 150 males and 144 females. Triangular fuzzy number processing was then performed on the questionnaire data, and the average fuzzy numbers of all subjects for each adjective were calculated and presented in Table 7. Finally, the ranking of the seven samples on the four groups of adjectives was plotted using triangular fuzzy numbers, as shown in Fig 8.

**Table 7:** Fuzzy number averages of the 7 samples for the 4 groups of adjectives.

Serial number	Precious–Exquisite	Sporty–Smart	Trendy–Technological	Distinctive–Avant-garde
S_5_	(5.2 6.0 7.6)	(6.4 7.6 8.7)	(5.8 6.9 8.3)	(5.5 6.5 7.9)
S_18_	(5.0 5.9 7.5)	(5.9 7.1 8.4)	(5.5 6.6 8.1)	(5.2 6.2 7.8)
S_22_	(5.2 6.2 7.8)	(5.5 6.5 8.1)	(5.4 6.4 8.0)	(5.2 6.1 7.7)
S_29_	(5.5 6.6 8.0)	(5.5 6.7 8.1)	(5.6 6.7 8.1)	(5.6 6.6 8.0)
S_34_	(5.6 6.7 8.2)	(5.7 6.9 8.3)	(5.5 6.7 8.1)	(5.5 6.6 8.1)
S_38_	(5.5 6.6 8.0)	(4.9 5.9 7.5)	(5.3 6.4 7.9)	(5.5 6.5 8.0)
S_41_	(5.5 6.6 8.0)	(4.9 6.0 7.5)	(5.2 6.2 7.7)	(5.4 6.2 7.7)

**Fig 9:**
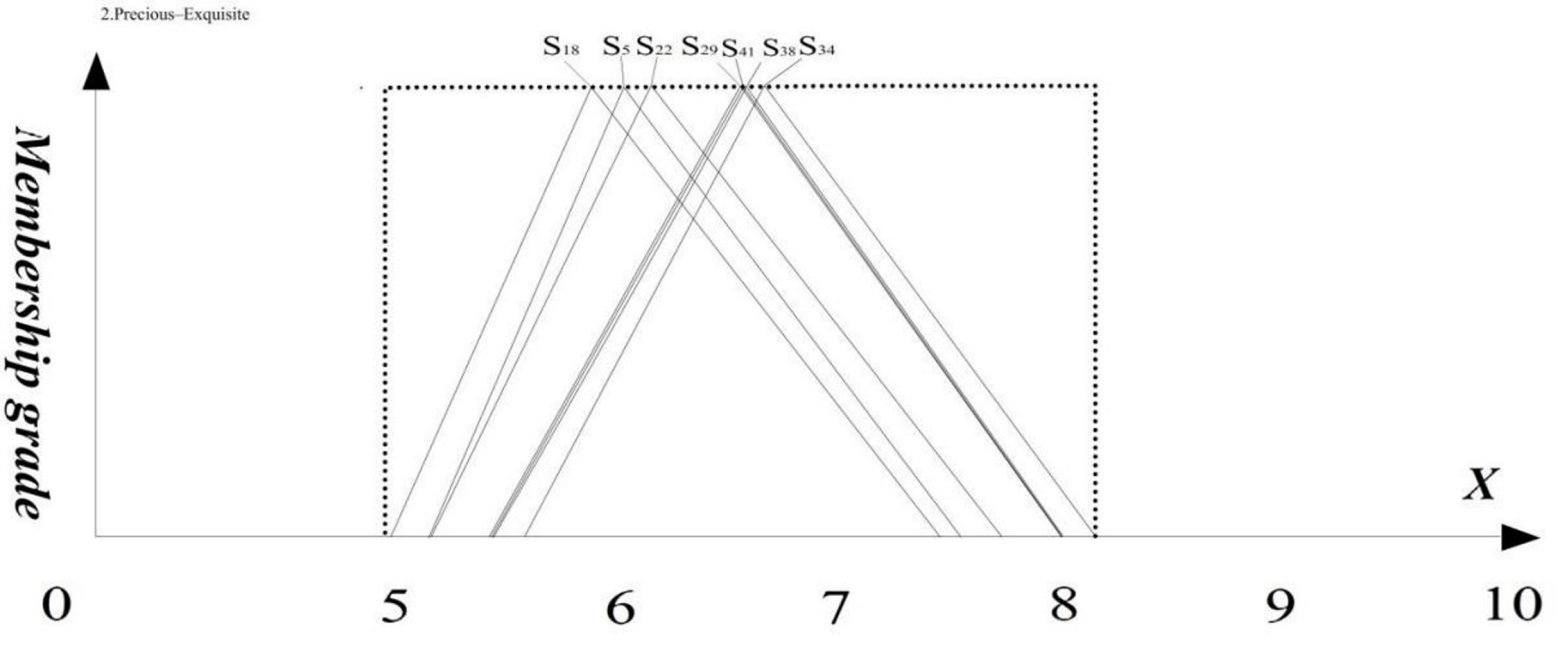
The triangular fuzzy numbers of 7 watch heads in the evaluation of "Sporty and Smart" visual imagery.

In order to obtain the performance of the seven samples on each visual image, we input the data in Table 6 into the *U_T_*(${\tilde{t}}_{i}$) equation to obtain the total utility value of the visual image of each sample on any adjective. Within the Sporty-Smart style, the fuzzy average number matrix of seven sports watches (Table 7), exemplified by the fuzzy average number of the Sporty-Smart S5, is displayed in the operation results presented in Fig 9.

Given $\tilde{t_{i}}$ = (*t*_*i*1_, *t*_*i*2_, *t*_*i*3_) = (6.4, 7.6, 8.7),*X*_*max*_ = 8.7,*X*_*min*_ = 4.9. Substitute the above values into the *U_T_*($\tilde{t_{i}}$) equation to obtain:

$${\text{U}}_{\text{T}}\left(\tilde{{\text{t}}_{i}}\right)=\left[\begin{array}{c} (8.7-4.9)/((8.7-4.9)+(8.7-7.6))+1\\ -(8.7-6.4)/((8.7-4.9)+(7.6-6.4)) \end{array}\right]/2=0.7607$$

By operating in the above manner, the absolute utility values of the seven watch heads in the four groups of adjectives were obtained and are shown in Table 8.

**Table 8:** Absolute utility values.

Wheel species	Precious—Exquisite	Sporty–Smart	Trendy—Technological	Distinctive—Avant-garde
S_5_	0.4142	0.7607*	0.6067*	0.5313
S_18_	0.5629*	0.6012	0.5547	0.5396
S_22_	0.5360	0.3997	0.4500	0.4579
S_29_	0.5360	0.5547	0.5596	0.5409*
S_34_	0.3871	0.6453	0.5396	0.4543
S_38_	0.5360	0.3842	0.4930	0.5208
S_41_	0.4543	0.5245	0.5011	0.4345

**Fig 10:**
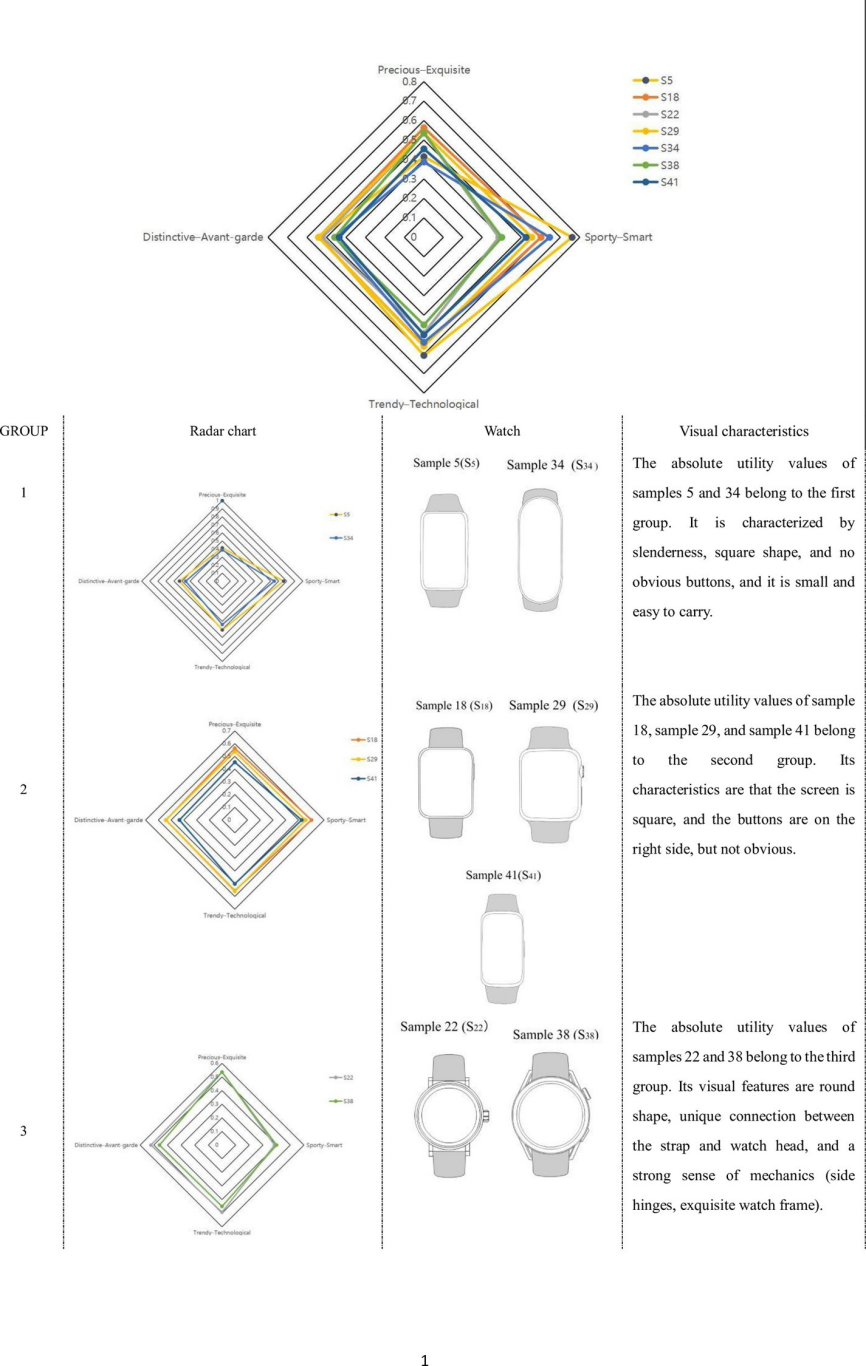
Radar chart.

Based on the research results, a radar chart showing the total utility value of the visual imagery of the seven samples in the four groups of adjectives was created (see Fig 10). Based on the spread of the seven samples across the four dimensions, the samples were categorized into three clusters.

The first group includes sample 5 and sample 34. The shape of these watches is slender and compact, with no extra decorative structure, which brings a strong sense of sportiness and simplicity to users, in line with the basic characteristics of sports watches. However, they may give users a slightly lower sense of value and differentiation, so precious and exquisite and distinctive and avant-garde adjectives received lower scores.

The second group includes sample 18, sample 29, and sample 41. The overall distribution in the four dimensions is relatively average, and the three samples have higher scores in the Sporty and Smart category, which is in line with the basic characteristics of sports watches. The visual characteristics include a relatively square screen and a stronger sense of volume, but more moderate than the first group, which can enhance the sense of value. Adding concealed buttons on the side can also enhance the technological sense.

The third group includes sample 22 and sample 38. Precious and exquisite, trendy and technological, and distinctive and avant-garde adjectives performed well, while Sporty and Smart received lower scores. The third group is different from the other groups in that precious and exquisite received higher scores. Its visual features include a round shape, a unique connection between the strap and watch head, and a strong sense of mechanics (side hinges, exquisite watch frame), which are important factors in creating feelings of preciousness and exquisiteness.

## Further analysis of a new application

Building upon the prior research conducted by Wei et al. which aimed to assist product designers in fulfilling consumers’ specific sensory experiences and expectations in perfume design using Kansei Engineering and Quantitative Theory I analysis [46]. this study extends the application to the realm of smart sports watches. By integrating the morphological element analysis from Kansei Engineering, this study designs a novel shape for smart sports watches.

In this study, a comprehensive analysis of the morphological elements of smart sports watches is conducted, as outlined in Table 9. The performance evaluation of seven samples across four distinct styles is summarized. As a result, the morphological elements corresponding to each of the four styles of smart sports watches are derived, as presented in Table 10.

**Table 9:** Morphological elements for smart sports watches.

Structure	1	2	3	4
**Screen (A)**	Round (A1)	Square with Rounded Corners (A2)	Rounded Rectangular (A3)	Long Oval (A4)
**Button (B)**	Rectangle with Rounded Corners Button (B1)	Flat Round Button (B2)	Round Button (B3)	No Buttons (B4)
**Knobs (C)**	Wide Round Knobs (C1)	Flat Round Knobs (C2)	No Knobs (C3)	
**Hinge (D)**	Hidden (D1)	Straight (D2)	Diagonal Cut (D3)	

**Table 10:** Characteristics of morphological elements in sports smart watch styles.

Style	Characteristics of Morphological Elements
**Precious—Exquisite**	Round (A1) / Square with Rounded Corners (A2) / Rectangle with Rounded Corners Button (B1) / Round Button (B3) / No Buttons (B4) / No Knobs (C3) / Hidden (D1) / Straight (D2) / Diagonal Cut (D3)
**Sporty—Smart**	Square with Rounded Corners (A2) / Rounded Rectangular (A3) / Rectangle with Rounded Corners Button (B1) / Flat Round Button (B2) / No Buttons (B4) / Flat Round Knobs (C2) / No Knobs (C3) / Hidden (D1)
**Trendy—Technological**	Rounded Rectangular (A3) / Rectangle with Rounded Corners Button (B1) / Flat Round Button (B2) / Flat Round Knobs (C2) / Hidden (D1)
**Distinctive—Avant-garde**	Square with Rounded Corners (A2) / Long Oval (A4) / Rectangle with Rounded Corners Button (B1) / No Buttons (B4) / Flat Round Knobs (C2) / Hidden (D1)

**Fig 11:**
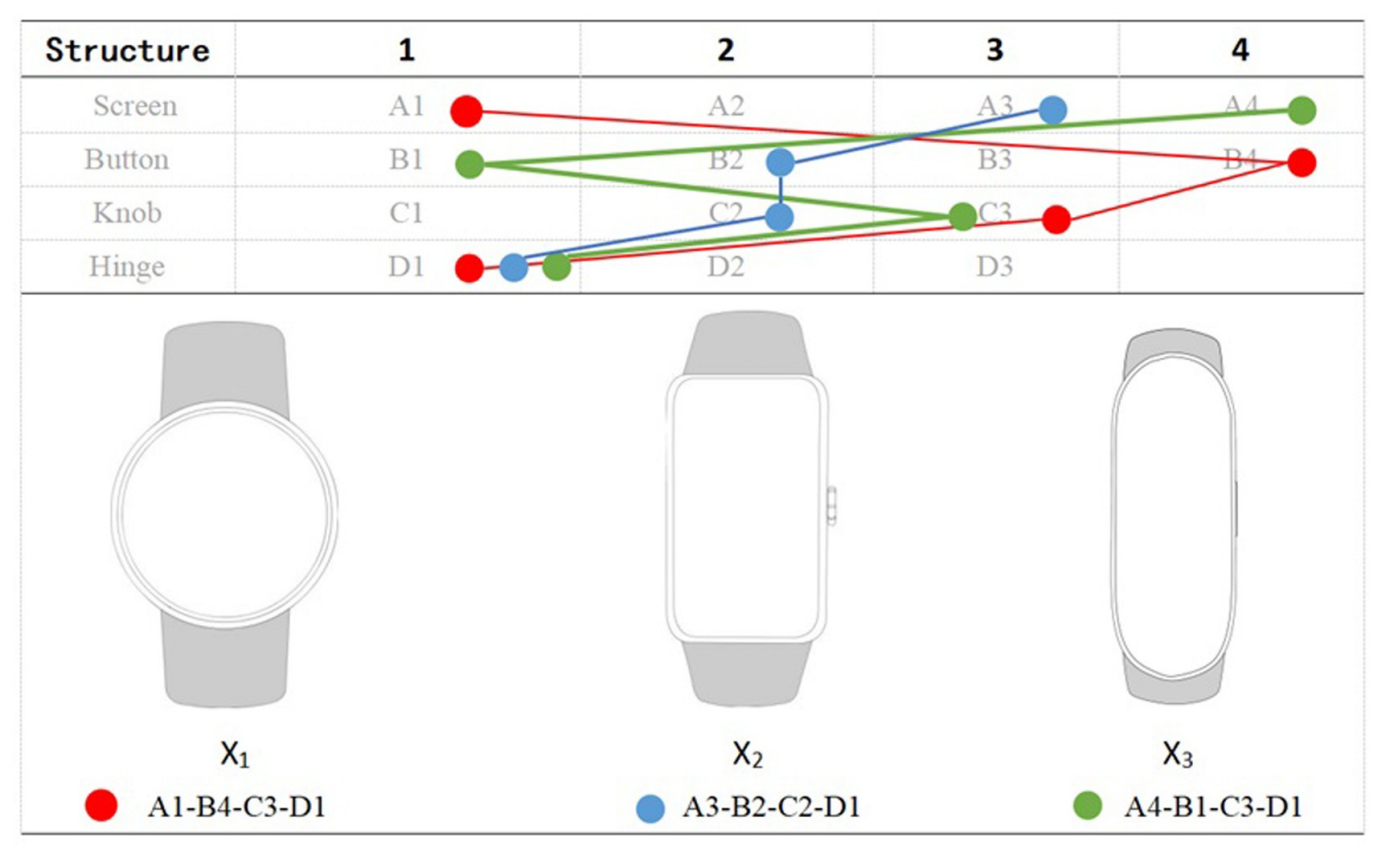
Three new design samples.

To further ascertain the representative and reliable of our findings, we fashioned three novel sports smartwatch models (Fig 11) based on the morphological elements of sports smart watches. These served as new samples for a stylistic survey, evaluating their performance across four distinct styles. Ultimately, out of 105 dispatched questionnaires, we retrieved 74 valid ones, discarding 31 for invalidity. The comparison analysis was subsequently conducted between the results of the new sample style survey and the morphological element representation in each sample (Table 10).

The survey data disclosed the score performances of the three new design samples across the four styles, i.e. Precious-Exquisite, Sporty-Smart, Trendy-Technological, and Distinctive-Avant-garde (Table 11).

**Table 11:** Ratings of three samples across four styles.

Serial number	Precious—Exquisite	Sporty—Smart	Trendy—Technological	Distinctive—Avant-garde
Sample X_1_	5.27*	4.68	4.39	2.91
Sample X_2_	4.36	5.18*	5.05	4.66
Sample X_3_	4.26	5.35*	4.14	4.95

(Note: An asterisk denotes the highest score)

Sample X_1_, the survey results revealed, leaned most toward the Precious-Exquisite style, scoring 5.27. Its morphological elements are composed of a round A1, no buttons B4, no knobs C3, and a hidden hinge D1. In terms of style, the morphological elements’ representation was as follows: Precious-Exquisite (4 elements) > Sporty-Smart (3 elements) > Trendy-Technological (1 element) = Distinctive-Avant-garde (1 element). A higher representation of morphological elements indicates closer alignment with the associated style. Consequently, the style of Sample X_1_’s dial appeals to users as predominantly Precious-Exquisite, consistent with the survey results.

Sample X_2_, as per survey outcomes, showed a significant preference for the Sporty-Smart and Trendy-Technological styles, scoring 5.18 and 5.05 respectively. Its morphological elements consist of a rounded rectangular A3, a flat round button B2, flat round knobs C2, and a hidden hinge D1. The representation of morphological elements across the four styles was as follows: Sporty-Smart (4 elements) ≧ Trendy-Technological (4 elements) > Distinctive-Avant-garde (2 elements) > Precious-Exquisite (0 elements). Therefore, the style of Sample X2’s dial gravitates toward the Sporty-Smart and Trendy-Technological styles, aligning with the survey findings.

Sample X_3_, the survey reported, veered most toward the Sporty-Smart and Distinctive-Avant-garde styles, scoring 5.35 and 4.95 respectively. The morphological elements comprised a long oval A4, a rectangle with rounded corners button B1, no knobs C3, and a hidden hinge D1. The representation of morphological elements across the styles was as follows: Sporty-Smart (3 elements) ≧ Distinctive-Avant-garde (3 elements) ≧ Precious-Exquisite (3 elements) > Trendy-Technological (2 elements). Thus, the style of Sample X3’s dial tends to resonate with the Sporty-Smart and Distinctive-Avant-garde styles, generally aligning with the survey findings.

By implementing the questionnaire survey on the three novel samples, we discovered that their performances across the four styles and their representation of style morphological elements were essentially consistent. This demonstrated a degree of representative and reliability in the preliminary research conclusions.

## Conclusions

This study utilized data collection, questionnaire surveys, reliability and validity analysis, factor analysis, and triangular fuzzy mathematics to explore the influence of seven watch head designs on consumers’ visual perception of smart sports watches. A total of 36 adjectives commonly used to describe watch heads were included in consumer questionnaires, and factor analysis was performed using SPSS 20 software to extract four groups of adjectives. A second consumer questionnaire survey and a triangular fuzzy operation were conducted to obtain the total utility values of the seven watch heads in terms of the four image adjective groups. The radar distribution map was then used to judge the influence of the watch head on the visual image of the smart sports watch.

In order to verify the stability and reliability of the research conclusions, we designed three new samples using the morphological elements of sports watches and conducted a questionnaire survey. The results found that the performance of the three samples in the four styles and the representation of style morphological elements were basically consistent, demonstrating that this research conclusion can provide a valuable reference in the design of sports smartwatch’s aesthetic appeal.

Upon categorization of the seven samples according to four adjective styles, we ultimately arrived at three distinct groups. We found that there were significant differences in the visual images of "Sporty and Smart" and "precious and exquisite" among the seven samples, while there was only a small difference between "distinctive and avant-garde" and "trendy and technological". Based on the grouping analysis of the seven samples, it is concluded that: the slim and compact shape without excessive decoration has a sense of sportiness and simplicity; the square shape combined with left and right buttons has a sense of sportiness and fashion; the unique connection between the round shape, the watch strap and the watch head, as well as the strong mechanical feeling, have a sense of value.

Our research results can be applied to the development and design of smart sports watches, allowing designers to better understand user perceptions of smart sports watches and avoid design deviation. Designers can choose appropriate watch heads based on different application scenarios and styles of different identity characteristics, thereby increasing user utilization and promoting the sustainable development of smart sports watches.

However, there are some limitations to this study. Firstly, the differences in aesthetic preferences of people with different lifestyles may lead to different cognitive results. As the data in this study are from China, the results are only applicable to the Chinese market environment. Secondly, due to the rapid replacement of smart sports watch products, researchers need to regularly replace research samples with the dominant products in the market to ensure the continuous accuracy of the research results. Lastly, since factors such as functionality, material, color, and others also influence consumer purchasing decisions, further research can be conducted on these aspects. Moreover, the improvement of the visual image of smart sports watches can still be evaluated in the later stage through more research methods, such as those using Big Data and artificial intelligence.
